# Effects of risk management plus predictive nursing on adolescents with non-suicidal self-injury behavior

**DOI:** 10.1590/1980-220X-REEUSP-2025-0379en

**Published:** 2026-03-23

**Authors:** Xiaowei Cui

**Affiliations:** 1 Beijing Huilongguan Hospital, Beijing, China.

**Keywords:** Adolescent, Risk Management, Nursing, Forecasting, Suicide, Self-Injurious Behavior

## Abstract

**Objective:**

To investigate the effects of risk management plus predictive nursing on adolescents with non-suicidal self-injury (NSSI) behavior.

**Method:**

Totally 122 adolescents with NSSI behavior hospitalized during February 2021 and May 2023 were included and categorized into an observation group (n = 61) and a control group (n = 61) according to the type of nursing care they received. Routine nursing was conducted for the control group, whereas risk management plus predictive nursing was performed for the observation group.

**Results:**

No intergroup difference was detected in self-injury behavior from the aspect of incidence rate before intervention (P > 0.05), while in contrast to that in the control group after intervention for 3, 6 and 12 months, it declined in the observation group (P < 0.05). Prior to intervention, no intergroup difference existed in the Social Dysfunction Screening Scale (P > 0.05), while the observation group presented a decrease in the score compared with the control group (P < 0.05). Significantly increased nursing satisfaction was obtained after intervention from the observation group (93.44%) in comparison to that from the control group (77.05%) (P < 0.05).

**Conclusion:**

Risk management plus predictive nursing can alleviate the NSSI behavior of adolescents.

## INTRODUCTION

At present, non-suicidal self-injury (NSSI) exhibits an increasing incidence among adolescents, posing serious threats to both physical and mental health^(1)^. Recent epidemiological surveys indicate that the prevalence of NSSI among adolescents typically ranges from 11.5% to 33.8% across international samples, with evidence suggesting a continued upward trend. Among Chinese youth, the pooled lifetime prevalence of NSSI has been estimated at approximately 25%^(2,3)^.

Adolescence, particularly between the ages of 13 and 18 years old, represents a critical developmental stage characterized by profound biological, psychological and social transitions. During this period adolescents experience rapid hormonal fluctuations, heightened emotional sensitivity and evolving self-identity which increase their vulnerability to emotional distress and maladaptive coping behaviors such as NSSI^(4)^. Psychosocial and environmental stressors including family conflict, academic pressure and social isolation further exacerbate this vulnerability^(5)^. Moreover, disparities in mental health resources and cultural norms between high- and low-income countries may influence the prevalence and coping patterns of NSSI^(6)^.

The harm of NSSI covers many dimensions. First of all (physical injury), intentional and repeated self-injury behaviors directly cause damage to body tissues. For example, common cuts may cause skin damage and bleeding, which, if not handled properly, will easily cause infection and even threaten life in severe cases. Bites and scratches can also cause local tissue damage and leave scars, not only affecting the appearance but also harming adolescents’ body image perception and self-confidence^(7)^.

Second (psychological impact), such behavior is often closely linked to complex psychological problems, such as depression and emotional regulation disorders. Self-injury behavior may become a way for adolescents to vent their inner pain and pressure to a certain extent, but such an unhealthy way cannot really solve the problem and will further worsen the psychological burden, creating a vicious circle of psychological problems, and even burying hidden dangers for long-term psychological diseases.

Furthermore, with regard to social functioning, non-suicidal self-injurious behavior has significant adverse effects on adolescents’ daily lives and social interactions. It may lead to declines in academic performance and difficulties with concentration, thereby impairing learning outcomes and future educational development. Moreover, adolescents engaging in NSSI may experience social withdrawal related to their behaviors in social contexts, increased tension in peer relationships, and difficulties integrating into typical social groups, which in turn negatively affects their social adaptability and future career development^(8)^. Therefore, NSSI among adolescents results in multifaceted and severe adverse consequences, highlighting the urgent need for effective nursing interventions to address this issue.

Predictive nursing, derived from proactive and risk-oriented nursing models, emphasizes early identification, dynamic monitoring, and anticipatory interventions aimed at preventing complications or behavioral deterioration. This approach is grounded in theories of anticipatory care and risk management, which advocate proactive assessment and individualized planning to address patients’ potential needs^(9)^. Recent research has demonstrated that predictive nursing can enhance psychological stability, treatment adherence, and safety outcomes among high-risk or psychiatric patients^(10)^.

However, few studies have examined the integration of predictive nursing with risk management strategies for adolescents with NSSI behavior. Based on this gap, the present study aimed to evaluate the effects of risk management plus predictive nursing on psychological status, self-injury frequency, and social adaptability among adolescents with NSSI. We hypothesized that this combined nursing approach would reduce the recurrence of NSSI and improve psychosocial outcomes.

## METHOD

### Subjects

This study was reviewed and approved by the ethics committee of our hospital (Approval No. 2021–58).

This study is a prospective controlled cohort study. A total of 122 adolescents with NSSI behavior hospitalized between February 2021 and May 2023 were recruited by convenience sampling. Eligible participants were initially identified by attending psychiatrists and psychological evaluations through routine inpatient assessments and medical record screening. Out of 138 eligible patients approached, 16 declined participation, resulting in 122 participants enrolled. Those who met the inclusion criteria were further evaluated using standardized diagnostic criteria for NSSI behavior. After confirming eligibility, written informed consent was obtained from both adolescents and their legal guardians prior to participation and implementation of nursing interventions.

According to the type of nursing care received during hospitalization, participants were categorized into the observation group (n = 61), who received risk management plus predictive nursing, and the control group (n = 61), who received routine ward-based nursing. Group allocation was determined by the actual nursing plan adopted. The two groups were comparable in baseline characteristics such as age, gender, and duration of illness (P > 0.05). The exclusion criteria were verified by two independent researches. The nursing staff and researchers who implemented the interventions received standardized training on predictive nursing and risk management before the study commenced. Outcome data were collected by independent assessors using standardized forms. The nursing protocol was reviewed and validated by two senior psychiatric nursing experts before implementation. Both groups received ward-based nursing within the same hospital environment, and follow-up assessments were conducted face-to-face during hospitalization or by scheduled phone interviews after discharge.

Inclusion criteria were as follows: (1) adolescents aged 13–18 years old, (2) those diagnosed with NSSI by professional psychiatrists or psychological evaluators according to the diagnostic guidelines of the ICD–11. The distinction between non-suicidal self-injury and suicide attempt was made based on the presence or absence of suicidal intent and confirmed by psychiatrists through clinical interviews. Only individuals who engaged in self-injury without suicidal intent were included^(11)^, (3) those with cognitive and communication skills who could understand and cooperate with nursing interventions and related assessments. Exclusion criteria involved: (1) adolescents with non-active and non-habitual NSSI behavior, defined as individuals with only one lifetime episode of NSSI that occurred more than 12 months before screening and no episodes within the past 6 months, or (2) those who were participating in other psychotherapy or drug clinical trials.

### Method for Control Group (Routine Nursing)

The patients’ vital signs were daily measured regularly, and regular rounds of wards were made to pay attention to signs of self-injury. Basic nursing was given, including keeping the ward clean, assisting patients in daily life, and planning a reasonable diet. Drug nursing was provided, including timely dispensing, observation of adverse reactions and explanation of precautions. In terms of psychological support, the patients should be daily communicated, at least twice a week, be comforted when faced with emotional instability, and be encouraged to hold a positive attitude to treatment and life.

### Method for Observation Group (Risk Management Plus Predictive Nursing)

Risk management comprised risk assessment, risk prevention measures, and emergency monitoring. During risk assessment, patient information was systematically collected, and psychological status and social factors were analyzed through communication with patients and their families, review of medical records, use of standardized psychological assessment tools, and consultations with teachers and classmates. Based on these data, a multidimensional risk assessment scale was established, classifying risk levels as low, moderate, or high. Risk prevention measures included weekly safety inspections of hospital wards, removal of potential self-injury items, installation of protective facilities, and routine patrols of wards and public areas every two hours. High-risk patients received psychological counseling twice weekly, cognitive behavioral therapy, and participation in psychological support group activities once per week.

In addition, monthly communication with patients’ family members was conducted, family training was provided, and social activity support was coordinated in collaboration with schools and community organizations. Risk assessments were repeated monthly to monitor emotional and behavioral changes and to adjust nursing interventions accordingly, and emergency response plans were established with semiannual drills.

Predictive nursing encompassed health education, psychological nursing, and life nursing. **Health education:** Monthly educational lectures were provided to patients and their families to introduce NSSI-related knowledge, accompanied by the distribution of informational brochures. Quarterly safety education campaigns were conducted to emphasize the hazards of self-injury. **Psychological nursing:** Nursing staff organized interactive activities once per week to enhance trust, recommended beneficial activities one to two times per week, and shared positive stories twice per month. **Life nursing:** A supportive sleep environment was created, healthy daily habits were encouraged, and patients were reminded to rest on time. Additionally, nursing staff encouraged patients to participate in daily activities according to their physical condition and assisted them in developing individualized activity plans.

This integrated nursing model was expected to achieve effective intervention outcomes in adolescents with NSSI behaviors.

### Evaluation of Outcomes

Incidence rate of self-injury behavior: Patients with NSSI behavior were counted over a given period of time (before intervention, and after 3, 6 and 12 months of intervention) in the two groups, and its incidence rate was calculated: (number of patients with NSSI behavior /total number of patients) ×100%.

Psychological state: Patients in both groups underwent quantitative assessment of depression degree by virtue of Self-rating Depression Scale (SDS)^(12)^, ranging from mild (53–62 points), moderate (63–72 points) to severe (≥73 points) depression. Besides, anxiety degree evaluation was completed by means of Self-rating Anxiety Scale (SAS) for both groups of patients^(13)^, classified as mild (50–59 points), moderate (60–69 points), and severe (≥70 points) anxiety. The evaluation was carried out before intervention, and after 3, 6 and 12 months of intervention.

Quality of life (QoL): The QoL of patients was evaluated using the Quality of Life Scale for Children and Adolescents (QLSCA) from various dimensions, each containing several items. Using the Likert scale method^(14,15)^, the total score was generally 1–5 points. A higher score signified better QoL. The scale was filled out by patients before and after intervention.

Social functional recovery: Social Dysfunction Screening Scale (SDSS) was employed to evaluate the social functional recovery of patients before and after intervention^(16)^.

Nursing compliance: It covered adherence to treatment plans and health instructions from caregivers, including non-compliance, partial, and full compliance. The calculation of compliance rate followed the rule of (number of full compliance + number of partial compliance)/total number ×100%.

Nursing satisfaction: A self-made questionnaire was used for the investigation of nursing satisfaction. The survey results included dissatisfied, basically satisfied, satisfied and very satisfied. The nursing satisfaction rate was calculated: (cases of very satisfied + satisfied + basically satisfied)/total cases ×100%.

### Statistical Analysis

Statistical analysis was accomplished through SPSS 26.0 software. The format of mean ± standard deviation (‾x±s) for expression, together with the *t*-test for intergroup comparison, was applied to measurement data. Count data were represented as percentage (%), and subjected to the chi-square test for intergroup comparisons. Differences with statistical significance were described using P < 0.05. Additionally, an age-based subgroup analysis was conducted to compare intervention effects between younger (13–15 years) and older (16–18 years) adolescents. Group differences were tested using independent-sample t tests or χ^2^ tests, and potential group × age interactions were evaluated using two-way ANOVA.

## RESULTS

### Incidence Rate of Self-Injury Behavior

No intergroup difference of statistical significance was detected in self-injury behavior from the aspect of incidence rate before intervention (P > 0.05), but in contrast to that in the control group following intervention for 3, 6 and 12 months, it declined in the observation group, and differences with statistical significance were observed (P < 0.05) (Table 1).

**Table 1:** Incidence rate of self-injury behavior – Beijing, China, 2022.

Group	Case (n)	Before intervention	After 3 months of intervention	After 6 months of intervention	After 12 months of intervention
Control	61	34 (55.73)	23 (37.70)	15 (24.59)	13 (21.31)
Observation	61	35 (47.94)	18 (29.50)	6 (19.35)	2 (3.27)
χ^2^		-	-	-	8.841
*P*		-	-	-	0.031

### Psychological State

Prior to intervention, SDS and SAS scores were not statistically significantly different in both groups (P > 0.05). Following intervention for 3, 6 and 12 months, however, significant drops in these scores emerged in the observation group versus the control group, and the differences were of statistical significance (P < 0.05) (Table 2).

**Table 2:** Psychological state – Beijing, China, 2022.

Group	SDS score				SAS score			
	Before intervention	After 3 months of intervention	After 6 months of intervention	After 12 months of intervention	Before intervention	After 3 months of intervention	After 6 months of intervention	After 12 months of intervention
Control (n = 61)	58.32 ± 6.18	53.25 ± 5.87	49.82 ± 5.26	46.75 ± 4.89	56.85 ± 5.92	52.38 ± 5.63	48.97 ± 5.12	45.62 ± 4.78
Observation (n = 61)	57.98 ± 6.25	48.12 ± 5.34	43.56 ± 4.89	39.28 ± 4.35	56.53 ± 5.88	46.25 ± 5.02	41.83 ± 4.65	37.56 ± 4.21
t	0.302	5.049	6.807	8.914	0.299	6.347	8.062	9.882
*P*	0.763	0.001	0.001	0.001	0.765	0.001	0.001	0.001

### Qol of Both Groups

The intergroup differences in the total score of QLSCA and the score in each dimension were not statistically significant before intervention (P > 0.05), but with the control group for comparison, they were raised in the observation group subsequent to intervention, showing differences of statistical significance (P < 0.05) (Table 3).

**Table 3:** QoL of both groups – Beijing, China, 2022.

Dimension	Time	Control group (n = 61)	Observation group (n = 61)	t/χ^2^	*P*
Physiological function	Before intervention	2.85 ± 0.52	2.82 ± 0.55	0.309	0.757
	After intervention	3.21 ± 0.48	3.68 ± 0.53	5.133	0.001
Psychological function	Before intervention	2.78 ± 0.49	2.75 ± 0.51	0.331	0.741
	After intervention	3.12 ± 0.51	3.59 ± 0.58	4.752	0.001
Social function	Before intervention	2.69 ± 0.53	2.66 ± 0.55	0.306	0.759
	After intervention	3.05 ± 0.45	3.48 ± 0.56	4.674	0.001
Environment	Before intervention	2.81 ± 0.47	2.78 ± 0.48	0.348	0.727
	After intervention	3.18 ± 0.53	3.62 ± 0.61	4.252	0.001
Total score	Before intervention	11.13 ± 1.91	11.01 ± 2.09	0.331	0.741
	After intervention	12.56 ± 1.97	14.37 ± 2.28	4.691	0.001

### Social Functional Recovery

Before intervention, the two groups exhibited no difference of statistical significance in the SDSS score (P > 0.05). Moreover, the observation group presented a decrease in the score relative to the control group, and a difference of statistical significance was detected (P < 0.05) (Table 4).

**Table 4:** SDSS scores – Beijing, China, 2022.

Group	Case (n)	Before intervention	After intervention
Control	61	15.32 ± 3.14	10.54 ± 2.39
Observation	61	15.47 ± 3.24	7.27 ± 2.06
t		0.259	8.094
*P*		0.795	0.001

### Nursing Compliance

The observation group presented a significantly elevated nursing compliance rate, with the control group as the contrast (86.88% *vs.* 70.49%), exhibiting a difference with statistical significance (P < 0.05) (Table 5).

**Table 5:** Nursing compliance – Beijing, China, 2022.

Group	Case (n)	Full compliance	Partial compliance	Non-compliance	Compliance rate
Control	61	20 (32.79)	23 (37.70)	18 (29.51)	70.49
Observation	61	32 (52.46)	21 (34.43)	8 (13.11)	86.88
χ^2^					6.706
*P*					0.035

### Satisfaction With Nursing

Significantly improved nursing satisfaction was obtained subsequent to intervention from the observation group (93.44%) contrasted with the control group (77.05%), where a difference showing statistical significance was observed (P < 0.05) (Table 6).

**Table 6:** Nursing satisfaction – Beijing, China, 2022.

Group	Case (n)	Very satisfied	Satisfied	Basically satisfied	Dissatisfied	Satisfaction rate
Observation	61	25 (40.98)	20 (32.79)	12 (19.67)	4 (6.56)	93.44
Control	61	15 (24.59)	16 (26.23)	16 (26.23)	14 (22.95)	77.05
χ^2^						9.071
*P*						0.028

### Age Subgroup Analysis

Participants were stratified into younger (13–15 years) and older (16–18 years) adolescents. The age distribution was comparable between groups (χ^2^ = 1.18, P = 0.277). Subgroup analysis showed that the observation group had significantly lower SDS and SAS scores than the control group in both age subgroups (P < 0.001). The incidence of self-injury also showed a decreasing trend in both subgroups, although the differences did not reach statistical significance (P = 0.076–0.210). Two-way ANOVA revealed no significant group × age interaction (P = 0.47), indicating that the intervention effect was consistent across age subgroups (Table 7).

**Table 7:** Subgroup analysis by age – Beijing, China, 2022.

Outcome	Age group	Control (n)	Observation (n)	Control value	Observation value	t/χ^2^	P
SDS (score)	13–15 years	35	28	46.54 ± 4.99	39.38 ± 4.50	5.837	<0.001
	16–18 years	26	33	47.15 ± 4.91	39.22 ± 4.37	6.172	<0.001
SAS (score)	13–15 years	35	28	45.11 ± 4.50	38.08 ± 4.26	6.175	<0.001
	16–18 years	26	33	46.60 ± 5.36	37.25 ± 4.26	6.887	<0.001
Self-injury incidence (%)	13–15 years	35	28	25.0%	8.7%	1.571	0.210
	16–18 years	26	33	14.3%	0.0%	3.142	0.076

## DISCUSSION

This prospective controlled cohort study investigated the effects and clinical value of combining risk management with predictive nursing in 122 adolescents with non-suicidal self-injury (NSSI). The findings demonstrated that this integrated nursing approach significantly reduced the frequency of self-injury behaviors while improving psychological status, quality of life (QoL), nursing satisfaction, social functioning recovery, and nursing compliance among adolescents with NSSI.

The results of the present study showed that, compared with the control group, the observation group exhibited a significantly lower number of self-injury behaviors following intervention. This finding suggests that the combination of risk management and predictive nursing can effectively identify and intervene in risk factors associated with NSSI, thereby reducing the incidence of self-injury behaviors. Through comprehensive risk assessment—including systematic collection of patient information, evaluation of self-injury risk factors, and development of a structured risk assessment scale—risk management enables accurate identification of patients’ self-injury risk levels. Based on this assessment, the implementation of multidimensional risk prevention strategies, such as environmental safety management, psychological intervention and support, health education, and the establishment of emergency response mechanisms, contributes to reducing the incidence of self-injury behaviors^(17)^. Predictive nursing further enhances prevention by continuously observing and evaluating patients’ conditions, promptly identifying emotional fluctuations, behavioral changes, and psychological needs, and enabling the early implementation of nursing interventions to prevent the occurrence of self-injury behaviors^(18)^.

Compared with the control group, adolescents in the observation group demonstrated significant reductions in Self-Rating Depression Scale (SDS) and Self-Rating Anxiety Scale (SAS) scores after intervention, indicating that the combined nursing model effectively alleviated depression and anxiety in adolescents with NSSI. Establishing a positive nurse–patient relationship and providing psychological counseling and cognitive reconstruction help patients recognize and modify negative cognitive patterns and maladaptive thinking habits, improve emotional regulation abilities, and relieve depressive and anxious symptoms^(19)^.

Furthermore, both the total score and dimensional scores of the Quality of Life Scale for Children and Adolescents (QLSCA) increased significantly in the observation group compared with the control group, suggesting that risk management combined with predictive nursing effectively improves QoL in adolescents with NSSI. Life nursing and rehabilitation nursing, as important strategies for enhancing QoL, facilitate recovery from functional impairments by ensuring adequate sleep, balanced nutrition, and appropriate physical activity, as well as by promoting wound healing and functional rehabilitation training^(20)^. Social function rehabilitation focuses on helping patients gradually restore and enhance social abilities, enabling smoother reintegration into social life, improved interpersonal communication, and greater social adaptability, thereby contributing to better overall QoL^(21)^.

In addition, the observation group achieved higher levels of nursing compliance and nursing satisfaction than the control group, indicating that the combined nursing approach enhances patients’ understanding of and cooperation with nursing care, thereby improving both nursing quality and effectiveness. Individualized nursing plans, strong nurse–patient relationships, and effective psychological intervention and support address patients’ specific needs and increase satisfaction with care. Improved nursing compliance further facilitates the smooth implementation of nursing measures, thereby amplifying nursing outcomes. Risk management combined with predictive nursing constitutes a comprehensive and systematic nursing model encompassing risk assessment, risk prevention, condition monitoring, and targeted nursing interventions.

This approach provides holistic care for adolescents with NSSI, addresses patient needs at multiple levels, and enhances overall nursing effectiveness^(22)^. For example, for patients experiencing depression, psychological nursing goals may focus on improving emotional status and self-confidence through regular counseling, encouragement of outdoor activities, and development of personal interests^(23)^. As a preventive and forward-looking approach, predictive nursing enables early identification of factors contributing to self-injury behaviors and the timely implementation of corresponding preventive measures^(24)^. Through ongoing observation and evaluation, predictive nursing facilitates early detection of emotional changes, behavioral expressions, and psychological needs, allowing for proactive interventions to prevent the recurrence of self-injury behaviors^(25)^. Overall, risk management provides the foundation and guidance for predictive nursing through structured assessment and preventive strategies, while predictive nursing reduces the frequency of self-injury behaviors through continuous observation and timely intervention. Together, these approaches are complementary and provide comprehensive and effective nursing services for adolescents with NSSI^(26)^.

Compared with previous interventions that primarily focused on cognitive behavioral therapy, routine psychological counseling, or crisis management^(27)^, the present study proposes a more proactive and multidimensional nursing model that integrates continuous risk monitoring with anticipatory nursing interventions. Unlike traditional approaches that intervene only after self-injury has occurred, predictive nursing enables early detection of subtle emotional and behavioral changes, assessment of recurrence risk, and implementation of preventive measures in advance, thereby enhancing early identification, individualized support, and overall safety management for adolescents at risk of self-harm^(28)^.

The intervention also demonstrates good feasibility for broader clinical and community application, as it can be adapted to psychiatric wards, school-based programs, and community mental health services^(29)^. With appropriate institutional support, implementation primarily requires standardized nurse training in risk identification, psychological assessment, communication skills, and emergency response, which can be incorporated into existing psychiatric nursing curricula or continuing education programs to ensure consistent and safe practice. Consequently, the integration of risk management and predictive nursing offers a practical and innovative framework for improving the quality and continuity of adolescent mental health care, providing new perspectives on early intervention and rehabilitation for adolescents with NSSI across diverse healthcare and educational settings.

Several limitations should be acknowledged. This was a single-center study with a relatively small sample size, which may limit the generalizability of the findings. The observation period was relatively short, and long-term outcomes were not evaluated. Additionally, psychological and behavioral data were primarily self-reported, which may be subject to response bias. Despite these limitations, this study contributes valuable evidence to the field of nursing by proposing an integrated framework combining risk management and predictive nursing. The findings provide practical guidance for early identification and proactive intervention in adolescents with NSSI, supporting the advancement of psychiatric nursing toward a preventive and holistic care model.

## CONCLUSION

In conclusion, risk management plus predictive nursing, as a comprehensive, systematic, personalized, targeted, preventive, prospective, synergistic and integrated nursing mode, can provide comprehensive and effective nursing services for adolescents with NSSI behavior. In the future, larger-sample, longer-time and multicenter studies are required to more fully assess risk management plus predictive nursing for its effect and value on adolescents with NSSI behavior.

## Data Availability

The entire dataset supporting the results of this study is available upon request to the corresponding author.
