# Supplementary material for: Effects of risk management plus predictive nursing on adolescents with non-suicidal self-injury behavior
Source: Rev Esc Enferm USP. 2026 Mar 23;60:e20250379. doi: 10.1590/1980-220X-REEUSP-2025-0379en (PMC13008363; doi:10.1590/1980-220X-REEUSP-2025-0379en)
Supplement: Translation

# Translation: Efectos de la gestión de riesgos combinada con enfermería predictiva en adolescentes con conducta autolesiva no suicida

**DOI:** 10.1590/1980-220X-REEUSP-2025-0379es

**Authors:** Xiaowei Cui

**Affiliations:** 1 Beijing Huilongguan Hospital, Beijing, China.

**Keywords:** Adolescente, Gestión de Riesgos, Enfermería, Predicción, Suicidio, Conducta Autodestructiva

## Abstract

**Objetivo:**

Investigar los efectos de la gestión de riesgos combinada con la enfermería predictiva en adolescentes con conducta autolesiva no suicida (ALNS).

**Método:**

Se incluyeron 122 adolescentes con conducta autolesiva de ALNS hospitalizados entre febrero de 2021 y mayo de 2023, categorizados en un grupo de observación (n = 61) y un grupo control (n = 61) de acuerdo con el tipo de cuidado de enfermería recibido. El grupo control recibió cuidados de enfermería de rutina, mientras que el grupo de observación recibió gestión de riesgos combinada con la enfermería predictiva.

**Resultados:**

No hubo diferencia entre los grupos en la incidencia de conducta autolesiva antes de la intervención (P > 0,05), mientras que, tras 3, 6 y 12 meses de intervención, se observó una reducción de la incidencia en el grupo de observación en comparación con el grupo control (P < 0,05). Antes de la intervención, no hubo diferencia entre los grupos en la Escala de Cribado de Disfunción Social (P > 0,05), mientras que el grupo de observación presentó una disminución de la puntuación en comparación con el grupo control (P < 0,05). Se observó un aumento significativo de la satisfacción con los cuidados de enfermería después de la intervención en el grupo de observación (93,44%) en comparación con el grupo control (77,05%) (P < 0,05).

**Conclusión:**

La gestión de riesgos aliada a la enfermería predictiva puede atenuar la conducta autolesiva no suicida en adolescentes.

## INTRODUCCIÓN

En la actualidad, las autolesiones no suicidas (non-suicidal self-injuries-NSSI) muestran una incidencia creciente entre los adolescentes, lo que supone una grave amenaza para su salud física y mental^(1)^. Encuestas epidemiológicas recientes indican que la prevalencia de las NSSI entre los adolescentes oscila normalmente entre el 11,5% y el 33,8% en muestras internacionales, con datos que sugieren una tendencia al alza continuada. Entre los jóvenes chinos, la prevalencia combinada de NSSI a lo largo de la vida se ha estimado en aproximadamente el 25%^(2,3)^.

La adolescencia, especialmente entre los 13 y los 18 años, representa una etapa crítica del desarrollo caracterizada por profundas transiciones biológicas, psicológicas y sociales. Durante este periodo, los adolescentes experimentan rápidas fluctuaciones hormonales, una mayor sensibilidad emocional y una identidad propia en evolución, lo que aumenta su vulnerabilidad al malestar emocional y a comportamientos de afrontamiento inadaptados, como las NSSI^(4)^. Los factores de estrés psicosocial y ambiental, como los conflictos familiares, la presión académica y el aislamiento social, exacerban aún más esta vulnerabilidad^(5)^. Además, las disparidades en los recursos de salud mental y las normas culturales entre los países de ingresos altos y bajos pueden influir en la prevalencia y los patrones de afrontamiento de las NSSI^(6)^.

El daño de la NSSI abarca muchas dimensiones. En primer lugar (lesiones físicas), las conductas de autolesión intencionadas y repetidas causan daños directos a los tejidos corporales. Por ejemplo, los cortes comunes pueden causar daños en la piel y sangrado, lo que, si no se trata adecuadamente, puede provocar fácilmente infecciones e incluso poner en peligro la vida en casos graves. Las mordeduras y los arañazos también pueden causar daños locales en los tejidos y dejar cicatrices, lo que no solo afecta a la apariencia, sino que también perjudica la percepción de la imagen corporal y la confianza en sí mismos de los adolescentes^(7)^.

En segundo lugar (impacto psicológico), este tipo de comportamiento suele estar estrechamente relacionado con problemas psicológicos complejos, como la depresión y los trastornos de regulación emocional. El comportamiento autolesivo puede convertirse, en cierta medida, en una forma de que los adolescentes descarguen su dolor y presión internos, pero esta forma poco saludable no resuelve realmente el problema y empeora aún más la carga psicológica, creando un círculo vicioso de problemas psicológicos e incluso ocultando peligros para enfermedades psicológicas a largo plazo.

Además, en lo que respecta al funcionamiento social, el comportamiento autolesivo no suicida tiene efectos adversos significativos en la vida cotidiana y las interacciones sociales de los adolescentes. Puede provocar un descenso en el rendimiento académico y dificultades de concentración, lo que perjudica los resultados del aprendizaje y el desarrollo educativo futuro. Además, los adolescentes que se involucran en NSSI pueden experimentar un aislamiento social relacionado con sus comportamientos en contextos sociales, un aumento de la tensión en las relaciones con sus compañeros y dificultades para integrarse en grupos sociales típicos, lo que a su vez afecta negativamente a su adaptabilidad social y a su futuro desarrollo profesional^(8)^. Por lo tanto, el NSSI entre los adolescentes tiene consecuencias adversas graves y multifacéticas, lo que pone de relieve la urgente necesidad de intervenciones de enfermería eficaces para abordar esta cuestión.

La enfermería predictiva, derivada de modelos de enfermería proactivos y orientados al riesgo, hace hincapié en la identificación temprana, el monitoreo dinámico y las intervenciones anticipatorias destinadas a prevenir complicaciones o el deterioro del comportamiento. Este enfoque se basa en teorías de atención anticipatoria y gestión de riesgos, que abogan por una evaluación proactiva y una planificación individualizada para abordar las posibles necesidades de los pacientes^(9)^. Investigaciones recientes han demostrado que la enfermería predictiva puede mejorar la estabilidad psicológica, el cumplimiento del tratamiento y los resultados de seguridad entre los pacientes de alto riesgo o psiquiátricos^(10)^.

Sin embargo, pocos estudios han examinado la integración de la enfermería predictiva con estrategias de gestión de riesgos para adolescentes con comportamiento NSSI. Basándose en esta laguna, el presente estudio tuvo como objetivo evaluar los efectos de la gestión de riesgos más la enfermería predictiva sobre el estado psicológico, la frecuencia de autolesiones y la adaptabilidad social entre los adolescentes con NSSI. Nuestra hipótesis era que este enfoque de enfermería combinado reduciría la recurrencia de NSSI y mejoraría los resultados psicosociales.

## MÉTODO

### Sujetos

Este estudio fue revisado y aprobado por el comité de ética de nuestro hospital (n.º de aprobación 2021–58). Es un estudio prospectivo de cohortes controlado. Se reclutó a un total de 122 adolescentes con comportamiento NSSI hospitalizados entre febrero de 2021 y mayo de 2023 mediante un muestreo por conveniencia. Los participantes elegibles fueron identificados inicialmente por los psiquiatras responsables y mediante evaluaciones psicológicas a través de evaluaciones rutinarias de los pacientes hospitalizados y el análisis de los expedientes médicos. De los 138 pacientes elegibles contactados, 16 rechazaron participar, lo que dio como resultado 122 participantes inscritos. Aquellos que cumplían los criterios de inclusión fueron evaluados adicionalmente utilizando criterios diagnósticos estandarizados para el comportamiento de NSSI. Tras confirmar su elegibilidad, se obtuvo el consentimiento informado por escrito tanto de los adolescentes como de sus tutores legales antes de la participación y la implementación de las intervenciones de enfermería.

Según el tipo de atención de enfermería recibida durante la hospitalización, los participantes se clasificaron en el grupo de observación (n = 61), que recibió gestión de riesgos más enfermería predictiva, y el grupo de control (n = 61), que recibió enfermería rutinaria en la sala. La asignación de los grupos se determinó según el plan de enfermería real adoptado. Los dos grupos eran comparables en cuanto a características basales como la edad, el sexo y la duración de la enfermedad (P > 0,05). Los criterios de exclusión fueron verificados por dos investigadores independientes. El personal de enfermería y los investigadores que implementaron las intervenciones recibieron formación estandarizada sobre enfermería predictiva y gestión de riesgos antes de que comenzara el estudio. Los datos de los resultados fueron recolectados por evaluadores independientes utilizando formularios estandarizados. El protocolo de enfermería fue revisado y validado por dos expertos en enfermería psiquiátrica antes de su implementación. Ambos grupos recibieron cuidados de enfermería en la sala dentro del mismo entorno hospitalario, y las evaluaciones de seguimiento se realizaron en persona durante la hospitalización o mediante entrevistas telefónicas programadas después del alta.

Los criterios de inclusión fueron los siguientes: (1) adolescentes de entre 13 y 18 años, (2) aquellos diagnosticados con NSSI por psiquiatras profesionales o evaluadores psicológicos de acuerdo con las directrices diagnósticas de la CIE-11. La distinción entre autolesiones no suicidas e intentos de suicidio se realizó en función de la presencia o ausencia de intención suicida y fue confirmada por psiquiatras mediante entrevistas clínicas. Solo se incluyeron personas que se autolesionaban sin intención suicida^(11)^, (3) aquellas con habilidades cognitivas y de comunicación que podían comprender y cooperar con las intervenciones de enfermería y las evaluaciones relacionadas.

Los criterios de exclusión fueron: (1) adolescentes con comportamiento NSSI no activo y no habitual, definido como personas con un solo episodio de NSSI en toda su vida que se produjo más de 12 meses antes de la selección y sin episodios en los últimos 6 meses, o (2) aquellos que participaban en otras psicoterapias o ensayos clínicos con medicamentos.

### Método Para el Grupo de Control (Enfermería Rutinaria)

Se tomaban diariamente y de forma regular las constantes vitales de los pacientes y se realizaban rondas periódicas por las salas para prestar atención a los signos de autolesiones. Se proporcionaba atención de enfermería básica, lo que incluía mantener limpia la sala, ayudar a los pacientes en su vida diaria y planificar una dieta razonable. Se proporcionaba atención de enfermería farmacológica, lo que incluía la dispensación oportuna de medicamentos, la observación de reacciones adversas y la explicación de las precauciones. En cuanto al apoyo psicológico, se debía comunicar con los pacientes a diario, al menos dos veces por semana, consolarlos cuando se enfrentaban a inestabilidad emocional y animarlos a mantener una actitud positiva hacia el tratamiento y la vida.

### Método Para el Grupo de Observación (Gestión de Riesgos Más Cuidados Predictivos)

La gestión de riesgos comprendía la evaluación de riesgos, las medidas de prevención de riesgos y el monitoreo de emergencias. Durante la evaluación de riesgos, se recolectó sistemáticamente la información de los pacientes y se analizaron su estado psicológico y los factores sociales mediante la comunicación con los pacientes y sus familias, la revisión de los expedientes médicos, el uso de herramientas de evaluación psicológica estandarizadas y las consultas con profesores y compañeros de clase. A partir de estos datos, se estableció una escala de evaluación de riesgos multidimensional, que clasificaba los niveles de riesgo como bajos, moderados o altos. Las medidas de prevención de riesgos incluían inspecciones semanales de seguridad de las salas del hospital, la retirada de objetos que pudieran utilizarse para autolesionarse, la instalación de dispositivos de protección y rondas rutinarias de las salas y las zonas comunes cada dos horas. Los pacientes de alto riesgo recibían asesoramiento psicológico dos veces por semana, terapia cognitivo-conductual y participaban en actividades de grupos de apoyo psicológico una vez por semana.

Además, se mantuvo una comunicación mensual con los familiares de los pacientes, se impartió formación a las familias y se coordinó el apoyo a las actividades sociales en colaboración con las escuelas y las organizaciones comunitarias. Las evaluaciones de riesgo se repitieron mensualmente para monitorear los cambios emocionales y de comportamiento y ajustar las intervenciones de enfermería en consecuencia, y se establecieron planes de respuesta a emergencias con simulacros semestrales.

La enfermería predictiva abarcaba la educación sanitaria, la enfermería psicológica y la enfermería de vida. **Educación sanitaria:** Se impartían conferencias educativas mensuales a los pacientes y sus familias para presentarles conocimientos relacionados con las autolesiones, acompañadas de la distribución de folletos informativos. Se llevaban a cabo campañas trimestrales de educación sobre seguridad para enfatizar los peligros de las autolesiones. **Enfermería psicológica:** El personal de enfermería organizaba actividades interactivas una vez por semana para fomentar la confianza, recomendaba actividades beneficiosas una o dos veces por semana y compartía historias positivas dos veces al mes. **Enfermería de vida**: se creó un entorno propicio para el sueño, se fomentaron hábitos diarios saludables y se recordó a los pacientes que descansaran a tiempo. Además, el personal de enfermería animó a los pacientes a participar en actividades diarias según su condición física y les ayudó a desarrollar planes de actividades individualizados.

Se esperaba que este modelo de enfermería integrado lograra resultados de intervención eficaces en adolescentes con conductas de NSSI.

### Evaluación de Los Resultados

Tasa de incidencia de conductas de autolesión: se contabilizó el número de pacientes con conductas de autolesión durante un periodo de tiempo determinado (antes de la intervención y después de 3, 6 y 12 meses de intervención) en los dos grupos, y se calculó su tasa de incidencia: (número de pacientes con conductas de autolesión / número total de pacientes) × 100%.

Estado psicológico: los pacientes de ambos grupos se sometieron a una evaluación cuantitativa del grado de depresión mediante la Escala de Autoevaluación de la Depresión (Self-rating Depression Scale - SDS)^(12)^, que va desde la depresión leve (53–62 puntos), moderada (63–72 puntos) hasta la grave (≥73 puntos). Además, se completó la evaluación del grado de ansiedad mediante la Escala de Autoevaluación de la Ansiedad (Self-rating Anxiety - Scale SAS) para ambos grupos de pacientes^(13)^, clasificada como ansiedad leve (50–59 puntos), moderada (60–69 puntos) y grave (≥70 puntos). La evaluación se llevó a cabo antes de la intervención y después de 3, 6 y 12 meses de intervención.

Calidad de vida (Quality of Life - Qol): La QoL de los pacientes se evaluó utilizando la Escala de Calidad de Vida para Niños y Adolescentes (Quality of Life Scale for Children and Adolescents - QLSCA) desde varias dimensiones, cada una de las cuales contenía varios ítems. Utilizando el método de la escala Likert^(14,15)^, la puntuación total era generalmente de 1 a 5 puntos. Una puntuación más alta significaba una mejor QoL. La escala fue completada por los pacientes antes y después de la intervención.

Recuperación funcional social: Se empleó la Escala de Detección de Disfunción Social (Social Dysfunction Screening Scale - SDSS) para evaluar la recuperación funcional social de los pacientes antes y después de la intervención^(16)^.

Cumplimiento de los cuidados de enfermería: Abarcaba el cumplimiento de los planes de tratamiento y las instrucciones sanitarias de los cuidadores, incluyendo el incumplimiento, el cumplimiento parcial y el cumplimiento total. El cálculo de la tasa de cumplimiento siguió la regla de (número de cumplimientos totales + número de cumplimientos parciales)/número total × 100%.

Satisfacción con la atención de enfermería: se utilizó un cuestionario elaborado por nosotros mismos para investigar la satisfacción con la atención de enfermería. Los resultados de la encuesta incluyeron insatisfecho, básicamente satisfecho, satisfecho y muy satisfecho. La tasa de satisfacción con la atención de enfermería se calculó de la siguiente manera: (casos de muy satisfecho + satisfecho + básicamente satisfecho)/casos totales × 100%.

### Análisis Estadístico

El análisis estadístico se realizó mediante el software SPSS 26.0. Se aplicó a los datos de medición el formato de media ± desviación estándar (x ± s) para la expresión, junto con la prueba t para la comparación entre grupos. Los datos de recuento se representaron como porcentaje (%) y se sometieron a la prueba de chi cuadrado para las comparaciones entre grupos.

Las diferencias con significación estadística se describieron utilizando P < 0,05. Además, se realizó un análisis de subgrupos basado en la edad para comparar los efectos de la intervención entre los adolescentes más jóvenes (13–15 años) y los más mayores (16–18 años). Las diferencias entre grupos se comprobaron mediante pruebas t de muestras independientes o pruebas χ^2^, y las posibles interacciones entre grupos y edades se evaluaron mediante ANOVA bidireccional.

## RESULTADOS

### Tasa de Incidencia del Comportamiento Autolesivo

No se detectaron diferencias estadísticamente significativas entre los grupos en cuanto a la tasa de incidencia de conductas autolesivas antes de la intervención (P > 0,05), pero, a diferencia del grupo de control tras la intervención durante 3, 6 y 12 meses, en el grupo de observación se observó una disminución y se observaron diferencias estadísticamente significativas (P < 0,05) (Tabla 1).

**Tabla 1:** Tasa de incidencia de conductas autolesivas – Pekín, China, 2022.

Grupo	Casos (n)	Antes de la intervención	Después de 3 meses de intervención	Después de 6 meses de intervención	Después de 12 meses de intervención
Control	61	34 (55,73)	23 (37,70)	15 (24,59)	13 (21,31)
Observación	61	35 (47,94)	18 (29,50)	6 (19,35)	2 (3,27)
χ^2^		-	-	-	8,841
*P*		-	-	-	0,031

### Estado Psicológico

Antes de la intervención, las puntuaciones SDS y SAS no presentaban diferencias estadísticamente significativas en ambos grupos (P > 0,05). Sin embargo, tras la intervención durante 3, 6 y 12 meses, se observaron descensos significativos en estas puntuaciones en el grupo de observación frente al grupo de control, y las diferencias fueron estadísticamente significativas (P < 0,05) (Tabla 2).

**Tabla 2:** Estado psicológico – Pekín, China, 2022.

Grupo	Puntuación SDS				Puntuación SAS			
	Antes de la intervención	Después de 3 meses de intervención	Después de 6 meses de intervención	Después de 12 meses de intervención	Antes de la intervención	Después de 3 meses de intervención	Después de 6 meses de intervención	Después de 12 meses de intervención
Control (n = 61)	58,32 ± 6,18	53,25 ± 5,87	49,82 ± 5,26	46,75 ± 4,89	56,85 ± 5,92	52,38 ± 5,63	48,97 ± 5,12	45,62 ± 4,78
Observación (n = 61)	57,98 ± 6,25	48,12 ± 5,34	43,56 ± 4,89	39,28 ± 4,35	56,53 ± 5,88	46,25 ± 5,02	41,83 ± 4,65	37,56 ± 4,21
t	0,302	5,049	6,807	8,914	0,299	6,347	8,062	9,882
*P*	0,763	0,001	0,001	0,001	0,765	0,001	0,001	0,001

### Calidad de Vida de Ambos Grupos

Las diferencias entre los grupos en la puntuación total del QLSCA y en la puntuación de cada dimensión no fueron estadísticamente significativas antes de la intervención (P > 0,05), pero en comparación con el grupo de control, aumentaron en el grupo de observación tras la intervención, mostrando diferencias estadísticamente significativas (P < 0,05) (Tabla 3).

**Tabla 3:** Calidad de vida de ambos grupos – Pekín, China, 2022.

Dimensión	Tiempo	Grupo de control (n = 61)	Grupo de observación (n = 61)	t/χ^2^	*P*
Función fisiológica	Antes de la intervención	2,85 ± 0,52	2,82 ± 0,55	0,309	0,757
	Después de la intervención	3,21 ± 0,48	3,68 ± 0,53	5,133	0,001
Función psicológica	Antes de la intervención	2,78 ± 0,49	2,75 ± 0,51	0,331	0,741
	Después de la intervención	3,12 ± 0,51	3,59 ± 0,58	4,752	0,001
Función social	Antes de la intervención	2,69 ± 0,53	2,66 ± 0,55	0,306	0,759
	Después de la intervención	3,05 ± 0,45	3,48 ± 0,56	4,674	0,001
Entorno	Antes de la intervención	2,81 ± 0,47	2,78 ± 0,48	0,348	0,727
	Después de la intervención	3,18 ± 0,53	3,62 ± 0,61	4,252	0,001
Puntuación total	Antes de la intervención	11,13 ± 1,91	11,01 ± 2,09	0,331	0,741
	Después de la intervención	12,56 ± 1,97	14,37 ± 2,28	4,691	0,001

### Recuperación Funcional Social

Antes de la intervención, los dos grupos no mostraron diferencias estadísticamente significativas en la puntuación SDSS (P > 0,05). Además, el grupo de observación presentó una disminución en la puntuación en relación con el grupo de control, y se detectó una diferencia estadísticamente significativa (P < 0,05) (Tabla 4).

**Tabla 4:** Puntuaciones SDSS – Pekín, China, 2022.

Grupo	Casos (n)	Antes de la intervención	Después de la intervención
Control	61	15,32 ± 3,14	10,54 ± 2,39
Observación	61	15,47 ± 3,24	7,27 ± 2,06
t		0,259	8,094
*P*		0,795	0,001

### Cumplimiento de Cuidados

El grupo de observación presentó una tasa de cumplimiento de cuidados de enfermería significativamente elevada, en contraste con el grupo de control (86,88% *frente a* 70,49%), lo que muestra una diferencia con significación estadística (P < 0,05) (Tabla 5).

**Tabla 5:** Cumplimiento de los cuidados de enfermería – Pekín, China, 2022.

Grupo	Casos (n)	Cumplimiento total	Cumplimiento parcial	Incumplimiento	Tasa de cumplimiento
Control	61	20 (32,79)	23 (37,70)	18 (29,51)	70,49
Observación	61	32 (52,46)	21 (34,43)	8 (13,11)	86,88
χ^2^					6,706
*P*					0,035

### Satisfacción Con la Atención de Enfermería

Se obtuvo una mejora significativa en la satisfacción con la atención de enfermería tras la intervención del grupo de observación (93,44%) en comparación con el grupo de control (77,05%), donde se observó una diferencia estadísticamente significativa (P < 0,05) (Tabla 6).

**Tabla 6:** Satisfacción con la atención de enfermería – Pekín, China, 2022.

Grupo	Casos (n)	Muy satisfecho	Satisfecho	Básicamente satisfecho	Insatisfecho	Índice de satisfacción
Observación	61	25 (40,98)	20 (32,79)	12 (19,67)	4 (6,56)	93,44
Control	61	15 (24,59)	16 (26,23)	16 (26,23)	14 (22,95)	77,05
χ^2^						9,071
*P*						0,028

### Análisis Por Subgrupos de Edad

Los participantes se estratificaron en adolescentes más jóvenes (13–15 años) y mayores (16–18 años). La distribución por edades fue comparable entre los grupos (χ^2^ = 1,18, P = 0,277). El análisis por subgrupos mostró que el grupo de observación tenía puntuaciones SDS y SAS significativamente más bajas que el grupo de control en ambos subgrupos de edad (P < 0,001). La incidencia de autolesiones también mostró una tendencia a la baja en ambos subgrupos, aunque las diferencias no alcanzaron significación estadística (P = 0,076–0,210). El ANOVA bidireccional no reveló ninguna interacción significativa entre el grupo y la edad (P = 0,47), lo que indica que el efecto de la intervención fue consistente en todos los subgrupos de edad (Tabla 7).

**Tabla 7:** Análisis de subgrupos por edad – Pekín, China, 2022.

Resultado	Grupo de edad	Control (n)	Observación (n)	Valor de control	Valor de observación	t/χ^2^	P
SDS (puntuación)	13–15 años	35	28	46,54 ± 4,99	39,38 ± 4,50	5,837	<0,001
	16–18 años	26	33	47,15 ± 4,91	39,22 ± 4,37	6,172	<0,001
SAS (puntuación)	13–15 años	35	28	45,11 ± 4,50	38,08 ± 4,26	6,175	<0,001
	16–18 años	26	33	46,60 ± 5,36	37,25 ± 4,26	6,887	<0,001
Incidencia de autolesiones (%)	13–15 años	35	28	25,0	8,7	1,571	0,210
	16–18 años	26	33	14,3	0,0	3,142	0,076

## DISCUSIÓN

Este estudio prospectivo controlado de cohortes investigó los efectos y el valor clínico de combinar la gestión de riesgos con la enfermería predictiva en 122 adolescentes con autolesiones no suicidas (NSSI). Los resultados demostraron que este enfoque de enfermería integrado redujo significativamente la frecuencia de los comportamientos autolesivos, al tiempo que mejoró el estado psicológico, la calidad de vida (QoL), la satisfacción con la enfermería, la recuperación del funcionamiento social y el cumplimiento de los cuidados de enfermería entre los adolescentes con NSSI.

Los resultados del presente estudio mostraron que, en comparación con el grupo de control, el grupo de observación presentó un número significativamente menor de conductas de autolesión tras la intervención. Este hallazgo sugiere que la combinación de la gestión de riesgos y la enfermería predictiva puede identificar e intervenir eficazmente en los factores de riesgo asociados a las NSSI, reduciendo así la incidencia de las conductas de autolesión. A través de una evaluación integral de riesgos, que incluye la recolección sistemática de información sobre los pacientes, la evaluación de los factores de riesgo de autolesiones y el desarrollo de una escala estructurada de evaluación de riesgos, la gestión de riesgos permite identificar con precisión los niveles de riesgo de autolesiones de los pacientes. Sobre la base de esta evaluación, la aplicación de estrategias multidimensionales de prevención de riesgos, como la gestión de la seguridad ambiental, la intervención y el apoyo psicológicos, la educación sanitaria y el establecimiento de mecanismos de respuesta a emergencias, contribuye a reducir la incidencia de conductas autolesivas^(17)^. La enfermería predictiva mejora aún más la prevención al observar y evaluar continuamente el estado de los pacientes, identificar rápidamente las fluctuaciones emocionales, los cambios de comportamiento y las necesidades psicológicas, y permitir la implementación temprana de intervenciones de enfermería para prevenir la aparición de conductas autolesivas^(18)^.

En comparación con el grupo de control, los adolescentes del grupo de observación mostraron reducciones significativas en las puntuaciones de la Escala de Autoevaluación de la Depresión (SDS) y la Escala de Autoevaluación de la Ansiedad (SAS) después de la intervención, lo que indica que el modelo de enfermería combinado alivió eficazmente la depresión y la ansiedad en los adolescentes con NSSI. El establecimiento de una relación positiva entre el personal de enfermería y los pacientes, así como la prestación de asesoramiento psicológico y la reconstrucción cognitiva, ayudan a los pacientes a reconocer y modificar los patrones cognitivos negativos y los hábitos de pensamiento inadaptados, a mejorar sus capacidades de regulación emocional y a aliviar los síntomas depresivos y de ansiedad^(19)^.

Además, tanto la puntuación total como las puntuaciones dimensionales de la Escala de Calidad de Vida para Niños y Adolescentes (QLSCA) aumentaron significativamente en el grupo de observación en comparación con el grupo de control, lo que sugiere que la gestión de riesgos combinada con la enfermería predictiva mejora eficazmente la calidad de vida de los adolescentes con NSSI. La enfermería de vida y la enfermería de rehabilitación, como estrategias importantes para mejorar la calidad de vida, facilitan la recuperación de las deficiencias funcionales al garantizar un sueño adecuado, una nutrición equilibrada y una actividad física apropiada, así como al promover la cicatrización de heridas y el entrenamiento de rehabilitación funcional^(20)^. La rehabilitación de la función social se centra en ayudar a los pacientes a restaurar y mejorar gradualmente sus habilidades sociales, lo que les permite una reintegración más fluida en la vida social, una mejor comunicación interpersonal y una mayor adaptabilidad social, contribuyendo así a una mejor calidad de vida en general^(21)^.

Además, el grupo de observación alcanzó niveles más altos de cumplimiento y satisfacción con la enfermería que el grupo de control, lo que indica que el enfoque de enfermería combinado mejora la comprensión y la cooperación de los pacientes con los cuidados de enfermería, mejorando así tanto la calidad como la eficacia de la enfermería. Los planes de enfermería individualizados, las relaciones sólidas entre el personal de enfermería y los pacientes, y la intervención y el apoyo psicológicos eficaces abordan las necesidades específicas de los pacientes y aumentan la satisfacción con la atención. La mejora del cumplimiento de la enfermería facilita aún más la aplicación fluida de las medidas de enfermería, amplificando así los resultados de la misma. La gestión de riesgos combinada con la enfermería predictiva constituye un modelo de enfermería integral y sistemático que abarca la evaluación de riesgos, la prevención de riesgos, el monitoreo del estado y las intervenciones de enfermería específicas.

Este enfoque proporciona una atención holística a los adolescentes con NSSI, aborda las necesidades de los pacientes en múltiples niveles y mejora la eficacia general de la enfermería^(22)^. Por ejemplo, en el caso de los pacientes que sufren depresión, los objetivos de la enfermería psicológica pueden centrarse en mejorar el estado emocional y la confianza en sí mismos mediante el asesoramiento regular, el fomento de las actividades al aire libre y el desarrollo de intereses personales^(23)^. Como enfoque preventivo y prospectivo, la enfermería predictiva permite identificar tempranamente los factores que contribuyen a los comportamientos de autolesión y aplicar oportunamente las medidas preventivas correspondientes^(24)^. A través de la observación y la evaluación continuas, la enfermería predictiva facilita la detección temprana de cambios emocionales, expresiones conductuales y necesidades psicológicas, lo que permite intervenciones proactivas para prevenir la recurrencia de los comportamientos de autolesión^(25)^. En general, la gestión de riesgos proporciona la base y la orientación para la enfermería predictiva a través de evaluaciones estructuradas y estrategias preventivas, mientras que la enfermería predictiva reduce la frecuencia de los comportamientos de autolesión a través de la observación continua y la intervención oportuna. En conjunto, estos enfoques son complementarios y proporcionan servicios de enfermería integrales y eficaces para los adolescentes con NSSI^(26)^.

En comparación con las intervenciones anteriores, que se centraban principalmente en la terapia cognitivo-conductual, el asesoramiento psicológico rutinario o la gestión de crisis^(27)^, el presente estudio propone un modelo de enfermería más proactivo y multidimensional que integra la monitorización continua del riesgo con intervenciones de enfermería anticipatorias. A diferencia de los enfoques tradicionales que solo intervienen después de que se ha producido la autolesión, la enfermería predictiva permite la detección temprana de cambios emocionales y conductuales sutiles, la evaluación del riesgo de recurrencia y la aplicación de medidas preventivas por adelantado, lo que mejora la identificación temprana, el apoyo individualizado y la gestión general de la seguridad de los adolescentes en riesgo de autolesión^(28)^.

La intervención también demuestra una buena viabilidad para una aplicación clínica y comunitaria más amplia, ya que puede adaptarse a las salas psiquiátricas, los programas escolares y los servicios comunitarios de salud mental^(29)^. Con el apoyo institucional adecuado, la implementación requiere principalmente una formación estandarizada del personal de enfermería en identificación de riesgos, evaluación psicológica, habilidades de comunicación y respuesta a emergencias, que puede incorporarse a los planes de estudios de enfermería psiquiátrica existentes o a los programas de formación continua para garantizar una práctica coherente y segura. En consecuencia, la integración de la gestión de riesgos y la enfermería predictiva ofrece un marco práctico e innovador para mejorar la calidad y la continuidad de la atención de salud mental de los adolescentes, proporcionando nuevas perspectivas sobre la intervención temprana y la rehabilitación de los adolescentes con NSSI en diversos entornos de cuidados de salud y educativos.

Deben tenerse en cuenta varias limitaciones. Se trata de un estudio realizado en un solo centro con una muestra relativamente pequeña, lo que puede limitar la generalización de los resultados. El periodo de observación fue relativamente corto y no se evaluaron los resultados a largo plazo. Además, los datos psicológicos y conductuales fueron principalmente autoinformados, lo que puede estar sujeto a un sesgo de respuesta. A pesar de estas limitaciones, este estudio aporta una valiosa evidencia al campo de la enfermería al proponer un marco integrado que combina la gestión de riesgos y la enfermería predictiva. Los resultados proporcionan una guía práctica para la identificación temprana y la intervención proactiva en adolescentes con NSSI, lo que respalda el avance de la enfermería psiquiátrica hacia un modelo de atención preventiva y holística.

## CONCLUSIÓN

En conclusión, la gestión de riesgos más la enfermería predictiva, como modo de enfermería integral, sistemático, personalizado, específico, preventivo, prospectivo, sinérgico e integrado, puede proporcionar servicios de enfermería completos y eficaces a los adolescentes con comportamiento NSSI. En el futuro, se necesitarán estudios con muestras más amplias, de mayor duración y multicéntricos para evaluar más exhaustivamente la gestión de riesgos más la enfermería predictiva en cuanto a su efecto y valor en los adolescentes con comportamiento NSSI.

## Data Availability

El conjunto de datos que respaldan las conclusiones de este estudio está disponible previa solicitud al autor correspondiente.
